# The role of routine chest radiography after implantable venous access port catheter insertion under the guide of ultrasonography and fluoroscopy

**DOI:** 10.1186/s12962-022-00382-z

**Published:** 2022-08-23

**Authors:** Hamed Ghoddusi Johari, Mohammad Reza Saki, Amirhossein Erfani, Reza Shahriarirad, Keivan Ranjbar

**Affiliations:** 1 Vascular Surgery Department, Trauma Research Center, Shiraz University of Medical Sciences, Shiraz, Iran; 2 Thoracic and Vascular Surgery Research Center, Shiraz University of Medical Sciences, 71936-13311 Shiraz, Iran; 3 Student Research Committee, Shiraz University of Medical Sciences, Shiraz, Iran

**Keywords:** Vascular access devices, Pneumothorax, Hemothorax, Chest X-ray, Fluoroscopic, Ultrasonography

## Abstract

**Background:**

The objective of this study is the evaluation of routine chest radiography following the placement of Implantable venous access port catheter (IVAPC) central lines using combined ultrasound and fluoroscopic guidance by a vascular surgeon in the operating room.

**Material and method:**

A prospective study of 189 consecutive patients who underwent IVAPC central line insertion in the vascular surgery operating room from 2016 to 2019. Venipuncture was performed with an 18-gauge needle under the guidance of sonography in each case, and the access site was noted. The line position was confirmed by fluoroscopy following the procedure. Multiple tries for puncture and patients under 18 were excluded from our study. Routine radiography of chest was performed for all patients and pneumothorax, hemothorax, and catheter malposition were evaluated in each case.

**Results:**

There were 2 cases of asymptomatic pneumothorax, no cases of hemothorax, and all catheter tip positions were optimal or acceptable. The annual cost of chest radiography was 33,000,000IRR, 220 h of hospital and staff time, and 1.1 mSv radiation.

**Conclusion:**

In conclusion, when imaging guidance is used for IVAPC insertion by an experienced surgeon in a high-volume center, performing post-procedure routine chest radiography shows little benefit.

## Introduction

Since the introduction of chemotherapy, much attention has been paid to the achievement of an adequate means of venous access that is suitable for long-term use [1, 2]. The implantable of venous access port catheter (IVAPC) are valued devices for long-term intravenous treatment of cancer patients with cancer; however, the use and inserting of these devices are each linked with complications [3–6].

In 1992, Morris et al. reported their experience with radiological ultrasound and fluoroscopic guidance for the placement of port catheters. This study and subsequent studies showed low periprocedural complications and late complication rates, comparable to the standard surgical procedure. Radiological implantation usually uses jugular, subclavian or brachial veins as entry sites [7, 8, 9]. Radiological-interventional port catheter insertion under the guide of ultrasound and fluoroscopy is a procedure with an extremely high technical success rate and particularly low periprocedural complication rate. Ultrasound guidance is mainly responsible for the high technical success rate with zero periprocedural complication rates, fluoroscopy for exact catheter tip placement with a reduction of long-term complications. If the transjugular route is appointed as the catheter entry site, mechanical complications such as secondary migrations are rare and the progress of a pinch-off syndrome is unlikely. Patients and relatives are highly satisfied with the port system and experience more advantages than disadvantages [9–15].

Complications regarding port catheter insertions can be classified by the period of their appearance or by their underlying source. IVAPC insertion was first considered as a surgical procedure by means of entree through either the cephalic vein or the direct puncture of the jugular or subclavian vein, with complications related to either local or bloodstream infections [16].

In this study, we aim to evaluate the need for routine chest X-ray (CXR) after IVAPC under the guidance of ultrasonography and fluoroscopy.

## Material and method

This prospective study was conducted on 189 consecutive patients who underwent IVAPC central lines in the vascular surgery operating room from 2016 to 2019. The study was approved by the Ethics Committee of the Shiraz University of Medical Sciences (IR code: IR.sums.med.rec.1397.317). The patients’ records were anonymized and de-identified prior to analysis. The confidentiality of the details of the subjects was assured and written inform consent was obtained from the participants.

Venipuncture was performed with an 18-gauge needle under the guidance of sonography in each case, and the access site was noted. The line position was confirmed by fluoroscopy following the procedure. Routine radiography of chest was performed for all patients and pneumothorax, hemothorax and catheter malposition were evaluated in each case. Patients with catheter rout apart from the jugular vein, multiple tries for punctures, and under 18 years of age were excluded from our study.

Data were analyzed using SPSS (version 18; SPSS Inc., Chicago, IL, USA) to evaluate the frequency of each complication.

## Results

In our study, among the 189 enrolled patients, 116 were male and 73 were female. The rates of Hemothorax, Pneumothorax and the positioning of the catheter were evaluated in our study. Among the 189 patients, all of the patients had acceptable positioning, 2 (1.05%) developed mild pneumothorax and 0 (0%) developed Hemothorax. Table 1 demonstrates the frequency of post-IVAPC insertion complications.

**Table 1:** Frequency of Implantable venous access port catheter insertion complications

Variable		Frequency (%)	
		Male	Female
Population		116 (61.37)	73 (38.62)
Complication			
Pneumothorax		1 (0.86)	1 (0.86)
Hemothorax		0 (0)	0 (0)

Among the patients who developed pneumothorax, both were mild and didn’t need any further management. Both patients did not have symptoms related to their pneumothorax. One of the patients was a male lung cancer patient which also demonstrated pneumothorax in his previous images. The pneumothorax was also detected during our fluoroscopy. Reviewing previous imaging data revealed that pneumothorax was a chronic process. The second patient was a very cachexic female patient, in which the dome of the pleura was possibly pierced by the metal stilt used to pass the catheter, resulting in the patient’s pneumothorax. However, the patient was asymptomatic and did not require any further intervention or chest tube insertions, and was treated with conservative therapy.

In one of the patients which CXR showed haziness in favor of hemothorax, in which after a further investigation showed to be pleural effusion related to his disease which was also present in his previous chest radiographs.

### Cost-allocations evaluation

Based on our center’s database, during a 4-year period (2016–2019), a total of 438 cases of IVAPC were performed, calculating an approximate average annual rate of 110 cases. Based on our countries’ currency (Iran rial, IRR), we have estimated a cost of 300,000 IRR (80,000 IRR with insurance) for every single view frontal CXR, an average radiation dose of 0.1 mSv, and an average time of 2 h for each patient, including preparations, staff involvement, and coordination’s. The average time for CXR alone is also around 20 min for each patient. Based on these findings, an average hospital cost of 33,000,000 IRR, 220 h of staff involvement, and 11.1mSv radiation is used each year based on the current guidelines. Also, 36.7 h are spent on CXR alone.

## Discussion

The need for providing a mid to long term central venous catheter has increased with the use of chemotherapy drugs and long-term nutrition therapy. IVAPC has shown to be a safe procedure with minor complications. More than 15 million cases of central venous access take place in the United States annually with a complication rate of 5–19% [17].

Nowadays with the help of sonography guided approaches and the use of fluoroscopy, the rate of complications after catheter insertion has decreased. Surgeons used to perform IVAPC by dissecting a cephalic vein as a catheter entry site. Christoph M and colleagues reported the initial success rate of cephalic venous cutdown procedure of 80% and when using the radiological Seldinger technique the success rate increased up to 98.4–100% [10]. In 2011 another study done by Rabindranath KS showed a significant decrease in catheter insertion failure and time of procedure using sonography-guided hemodialysis catheter insertion [18]. A meta-analysis done by Randolph et al. demonstrated that using sonography-guided approaches for central venous catheterization of subclavian and jugular is preferred compared to landmark-based ones [19]. A standard practice to confirm the correct positioning of the port catheter and documentation of pneumothorax and hemithorax is to perform routine CXR. Many studies suggest performing routine chest X-rays only for patients who are clinically suspicious of hemithorax or pneumothorax, due to the low incidence of these complications [20–23]. In a study done by Burn and colleagues in central line placement in 3.844 patients, between 1994 and 1998, only 1.4% had pneumothorax in routine chest X-rays with only 0.1% of them not having any signs and symptoms [24].

In our study 2 patients (1.05%) developed pneumothorax and no cases of hemothorax after IVPAC which was similar to other studies done. The combined use of sonography and fluoroscopy in our study led to a high success rate of catheter positioning. It is worth mentioning that in our study, one of the patients whose CXR showed haziness in favor of hemothorax, which after further workup and evaluation showed to be disease-related pleural effusion which was also present in past chest radiographies. Therefore, we recommend the evaluation of past imaging and radiography in patients undergoing catheter insertion to lower the chance of being misled. Based on the mentioned patients, since the first patient was asymptomatic, and the patient present pneumothorax was also detected during fluoroscopy, a routine CXR was redundant. However, in the second patient, we suspected pleural injury during catheterization X-ray was required. However, these findings are in line with our results which demonstrate the excessiveness of routine chest-Xray performance in these patients, unless clinical suspicion or the physicians’ or surgeons’ judgment.

 Based on our center’s report, with an annular rate of 110 IVAPC patients, an average hospital cost of 33,000,000 IRR (≈ 300$ in 2019), 220 h of staff involvement, and 11.1 mSv radiation is used each year based on the current guidelines. Also, 36.7 h is spent on chest-X ray alone. Woodland et al. reported an annular rate of 155,000$ for unnecessary routine CXR after ultrasound-guided central venous line placement [25]. Based on a report by the World Health Organization (WHO), Iran Gross Domestic Product (GDP) per capita for 2020 was $2757, a 21.55% decline from 2019 ($3514), in which also had a 13.16% decline from 2018 [26]. Due to limited resources and staff, along with the high volume of our center, an adjustment in protocols regarding the necessity of CXR after IVAPC under the guidance of fluoroscopy.

With these findings in favor of low incidence of complications using combined fluoroscopy and sonography, the necessity of routine chest X-ray after IVAPC will be questioned.

Routine post-procedure chest X-rays in patients without signs and symptoms place an untenable burden on hospital resources. Factors contributing to higher costs of surgical implantations are higher costs of operating rooms, surgical assistants and operating nurses, and anesthesiologists stand by.

## Conclusion

By using sonography guided approaches and fluoroscopy for IVAPC, the incidence of adverse events such as hemothorax, pneumothorax, and catheter misplacement is reduced; therefore, post-procedure chest radiography in such cases seems unnecessary and excluding it will reduce hospital and patient’s costs and time consumption.

## Data Availability

SPSS data of the participant can be requested from the authors. Please write to the corresponding author if you are interested in such data.

## Abbreviations

CXR: Chest X-ray
IVAPC: Implantable venous access port catheter
